# Correction: *Genome Med* 15, 115 & *Genome Med* 16, 3

**DOI:** 10.1186/s13073-024-01343-2

**Published:** 2024-05-14

**Authors:** Ning Shen

**Affiliations:** 1 Department of Hepatobiliary and Pancreatic Surgery, First Affiliated Hospital, Zhejiang University School of Medicine, Hangzhou, 311121 Zhejiang Province China; 2 Department of Hepatobiliary and Pancreatic Surgery, First Affiliated Hospital & Liangzhu Laboratory, Zhejiang University School of Medicine, Hangzhou, 311121 China

**Correction:** **Genome Med 16, 3 (2024)**

**https://doi.org/10.1186/s13073-023-01274-4**

**Correction:** **Genome Med 15, 115 (2023)**

**https://doi.org/10.1186/s13073-023-01269-1**

The original publications of the below articles [1, 2] contained 2 errors. The incorrect and correct information is listed below. The original articles have been updated.

**Incorrect**

- **10.1186/s13073-023-01274-4 & 10.1186/s13073-023-01269-1**
  - ◦ 1 Department of Hepatobiliary and Pancreatic Surgery, First Affiliated Hospital, Zhejiang University School of Medicine, Hangzhou 311121, China
  - ◦ 2 Liangzhu Laboratory, Zhejiang University, 1369 West Wenyi Road, Hangzhou 311121, China
- **10.1186/s13073-023-01274-4**
  - ◦ This work was supported by the Leading Innovative and Entrepreneur Team Introduction Program of Zhejiang (No. 2021R01012) and the Starting Fund from Zhejiang University.

**Correct**

- **10.1186/s13073-023-01274-4 & 10.1186/s13073-023-01269-1**
  - ◦ 1 Department of Hepatobiliary and Pancreatic Surgery of the First Affiliated Hospital & Liangzhu Laboratory, Zhejiang University School of Medicine, Hangzhou, 311121, China
- **10.1186/s13073-023-01274-4**
  - ◦ This work was supported by the Key R&D Program of Zhejiang (No.2024SSYS0022), the Leading Innovative and Entrepreneur Team Introduction Program of Zhejiang (No. 2021R01012).
